# Esketamine modulates postoperative biochemical markers of oxidative stress, inflammation, and immune dysregulation in laparoscopic colorectal cancer surgery

**DOI:** 10.5937/jomb0-56862

**Published:** 2025-08-21

**Authors:** Peng Chen, Kun Qian, Kairun Zhu, Lu Xiaoqing, Liu Dajin

**Affiliations:** 1 Shanghai Jiao Tong University School of Medicine, Suzhou Kowloon Hospital, Department of Anesthesiology, Suzhou, China; 2 The Affiliated Huai'an Hospital of Xuzhou Medical University, Department of Joint Surgery, Huai'an, China

**Keywords:** antioxidant enzymes, inflammatory cytokines, immune biomarkers, perioperative biochemistry, esketamine, colorectal surgery, antioksidativni enzimi, inflamatorni citokini, imuni biomarkeri, perioperativna biohemija, esketamin, kolorektalna hirurgija

## Abstract

**Background:**

Laparoscopic colorectal cancer surgery, while minimally invasive, induces systemic oxidative stress, inflammation, and immune dysfunction through surgical trauma and anesthesia-related stress. Esketamine, an NMDA receptor antagonist with antioxidant and anti-inflammatory properties, may mitigate these biochemical perturbations. This study evaluated esketamine's effects on serum biomarkers of oxidative stress (glutathione, catalase, malondialdehyde, superoxide dismutase), inflammatory mediators (TNF-a, CRP IL-6), and T lymphocyte subsets in patients undergoing laparoscopic colorectal cancer resection.

**Methods:**

In this randomized controlled trial, 150 stage I-II colorectal cancer patients were allocated to esketamine (0.25 mg/kg bolus + 0.12 mg/kg/h infusion) or control (saline) groups during standardized anesthesia. Preand postoperative serum levels of oxidative stress markers (GSH, CAT, MDA, SOD), inflammatory cytokines (TNF-a, CRP IL-6), and immune cell subsets (CD3+, CD4+, CD8+, CD4+/CD8+ ratio) were quantified via ELISA and flow cytometry. Statistical analysis compared intergroup differences using t-tests and chi-square tests.

**Results:**

Postoperatively, the esketamine group exhibited significantly attenuated oxidative stress, with higher GSH (72.43± 6.63 vs. 60 .1 6± 5.57 mg/mL, P < 0.05), CAT (92.56± 8.31 vs. 82.81 ± 7.75 U/mL), and SOD (84.53± 8.02 vs. 69 .93± 7.05 nU/mL), alongside lower MDA (6.41± 0.52 vs. 9.52± 0.63 mmol/L). Pro-inflammatory cytokines were reduced (TNF-a: 4 0 .32 ± 4.84 vs. 54.37± 5.80 pg/mL; IL-6: 50.83± 5.05 vs. 82 .38± 8.46 pg/mL, P < 0.05). Immune function preservation was evident through elevated CD3+ (4 5 .1 8 ± 5 .0 1 % vs. 37 .05 ± 4.92% ) and CD4+ T cells (26.51 ±2.76% vs. 19.78± 2.09%), with a balanced CD4+/CD8+ ratio (1.12± 0.12 vs. 0.72± 0.09).

**Conclusions:**

Esketamine-based anesthesia significantly ameliorates postoperative oxidative damage, suppresses inflammatory cytokine release, and preserves cellular immune homeostasis, as evidenced by targeted biochemical and immunological analyses. These findings highlight esketamine's role in modulating perioperative biochemical pathways, potentially enhancing recovery in colorectal cancer surgery.

## Abstract (sr)

**Uvod:**

Laparoskopska hirurgija kolorektalnog karcinoma, iako je minimalno invazivna, izaziva sistemski oksidativni stres, upalu i imunološku disfunkciju kroz hiruršku traumu i stres povezan sa anestezijom. Esketamin, antagonist NMDA receptora sa antioksidativnim i antiinflamatornim svojstvima, može ublažiti ove biohemijske poremećaje. Ova studija je procenila efekte esketamina na serumske bio-markere oksidativnog stresa (glutation, katalaza, malondi-aldehid, superoksid dismutaza), inflamatorne medijatore (TNF-a, CRIP IL-6) i podgrupe T limfocita kod pacijenata koji su podvrgnuti laparoskopskoj laparoskopskoj boji karci-noma u boji.

**Metode:**

U ovom randomizovanom kontrolisanom ispitiva-nju, 150 pacijenata sa kolorektalnim karcinomom stadiju-ma I-II dodeljeno je esketamin (0,25 mg/kg bolus + 0,12 mg/kg/h infuzija) ili kontrolnoj (fiziološki rastvor) grupama tokom standardizovane anestezije. Prei postoperativni serumski nivoi markera oksidativnog stresa (GSH, CAT, MDA, SOD), inflamatornih citokina (TNF-a, CRP IL-6) i podskupova imunih ćelija (CD3+, CD4+, CD8+, CD4+/CD8+ odnos) su kvantifikovani putem ELISA testa i testa. Statistička analiza je upoređivala međugrupne razlike koriš-tenjem t-testova i hi-kvadrat testova.

**Rezultati:**

Postoperativno, grupa koja je primala esketamin pokazala je značajno smanjen oksidativni stres, sa višim GSH (72,43± 6,63 naspram 60,16± 5,57 mg/mL, P < 0,05), CAT (92,56±8,31 naspram 82,81 ±7,75 U/mL) i SOD (84,53± 8,02 naspram 69,93± 7,05 nU/mL), uz niži MDA (6,41± 0,52 naspram 9,52± 0,63 mmol/L). Proinflamatorni citokini su bili smanj'eni (TNF-a: 40,32± 4,84 naspram 54,37± 5,80 pg/mL; IL-6: 50,83± 5,05 naspram 82,38± 8,46 pg/mL, P < 0,05). Očuvanje imunološke funkcij'e bilo j'e evidentno kroz povišene CD3+ (45,18±5,01% naspram 37,05±4,92% ) i CD4+ T celij'e (26,51 ±2,76% naspram 19,78±2,09% ), sa uravnoteženim odnosom C D 4+/C d 8+ (1,12± 0,12 naspram 0,72± 0,09).

**Zaključak:**

Anestezija zasnovana na esketaminu značajno ublažava postoperativna oksidativna oštećenja, potiskuje otpuštanj'e inflamatornih citokina i čuva ćelij'sku imuno-lošku homeostazu, što j'e dokazano ciljanim biohemijskim i imunološkim analizama. Ovi nalazi naglašavaju ulogu eske-tamina u modulaciji perioperativnih biohemijskih puteva, potencijalno poboljšavajući oporavak u hirurgiji kolorek-talnog karcinoma.

## Introduction

Colorectal cancer (CRC), including colon cancer, is one of the most prevalent malignancies worldwide [1]. According to the World Health Organization, CRC ranks as the third most commonly diagnosed cancer and the second leading cause of cancer-related deaths globally, with nearly 2 million new cases and approximately 935,000 deaths reported annually [2] [3]. In China, the incidence of CRC has been steadily increasing, largely attributed to aging populations, urbanization, and shifts toward Westernized diets and sedentary lifestyles. Colon cancer, a subtype of CRC, has particularly become a major public health concern [4] [5]. Its etiology is multifactorial, involving genetic predisposition, environmental influences, and lifestyle factors. Mutations in oncogenes (e.g., KRAS) and tumor suppressor genes (e.g., APC, TP53), chronic inflammation, microbiota dysbiosis, and dietary carcinogens all play critical roles in the development of colon cancer [6] [7].

Treatment strategies for colon cancer have evolved significantly in recent decades. Non-surgical approaches, such as chemotherapy, radiotherapy, and targeted therapies, have improved overall survival and quality of life for patients with advanced disease. Surgical resection, however, remains the cornerstone of curative treatment for localized colon cancer. Among surgical techniques, laparoscopic colectomy has emerged as a preferred method due to its minimally invasive nature [8] [9] [10]. Laparoscopic surgery offers several advantages over traditional open surgery, including reduced postoperative pain, shorter hospital stays, faster recovery, and better cosmetic outcomes. However, it is not without complications, such as anastomotic leakage, infection, and thromboembolic events. Notably, laparoscopic surgery is associated with significant physiological stress responses, including oxidative stress and systemic inflammation, which can adversely impact patient recovery [11] [12]. Anesthesia plays a pivotal role in modulating these stress responses during laparoscopic colorectal cancer surgeries. Studies have shown that anesthetic regimens can influence oxidative stress levels, inflammatory reactions, and cellular immune function, making it a critical area of investigation.

Esketamine, an enantiomer of racemic ketamine, has recently garnered attention in anesthesia research due to its unique pharmacological properties [13] [14]. Esketamine, a potent enantiomer of ketamine, has gained attention in anesthesia research due to its NMDA receptor antagonism and immunomodulatory effects. Compared to propofol, remifentanil, and dexmedetomidine, esketamine uniquely combines analgesic, anti-inflammatory, and antioxidant properties, making it a promising perioperative agent [15]. This makes it a promising agent for use in multimodal anesthesia protocols. Recent studies have suggested that esketamine can exert anti-inflammatory and antioxidant effects, which may be particularly beneficial in patients undergoing laparoscopic colorectal cancer surgeries [16]. Esketamine-based anesthesia has been shown to attenuate stress responses, reduce pro-inflammatory cytokine release, and preserve cellular immune function, making it an attractive option for enhancing perioperative outcomes in these patients [17].

The present study aims to investigate the effects of esketamine-based anesthesia on oxidative stress, inflammatory responses, and cellular immune function in patients undergoing laparoscopic colorectal cancer surgery. Specifically, this study evaluates changes in serum levels of glutathione (GSH), catalase enzymes (CAT), malondialdehyde (MDA), superoxide dismutase (SOD), tumor necrosis factor-α (TNF-α), C-reactive protein (CRP), interleukin-6 (IL-6), and T lymphocyte subsets. By elucidating the mechanisms through which esketamine-based anesthesia influences these biomarkers, this study seeks to provide valuable insights into optimizing anesthetic strategies to improve surgical outcomes for patients with colon cancer.

## Materials and methods

### Participants

This study was conducted at our hospital between January 2020 and October 2024, enrolling a total of 150 patients diagnosed with colorectal cancer (CRC) who were scheduled to undergo laparoscopic colorectal cancer resection. The inclusion criteria were as follows: (1) diagnosis of colon cancer confirmed by colonoscopy and histopathological examination; (2) TNM stage l-ll colorectal cancer; (5) early-stage, solitary lesions of colon cancer; (4) patients who provided informed consent for participation. A power analysis was conducted to determine the appropriate sample size for this study. Based on previous studies evaluating the impact of anesthetic agents on oxidative stress and immune function, we estimated that a minimum detectable difference of 15% in oxidative stress biomarkers (e.g., GSH, SOD) between the esketamine and control groups would be clinically relevant. Using a significance level of α = 0.05 and a power of 80% (β = 0.20), the required sample size was calculated to be at least 65 patients per group. To account for potential dropouts or missing data, we increased the sample size to 75 patients per group, totaling 150 participants. This ensured adequate statistical power to detect meaningful differences in postoperative biochemical and immunological outcomes.

Exclusion criteria included: (1) patients with non-solitary colorectal cancer lesions; (2) patients who were not receiving their first treatment; (5) patients with comorbidities such as endocrine disorders, coagulation dysfunction, chronic infections, immune system diseases, or other malignancies; (4) patients with hypersensitivity or severe allergic reactions; (5) patients with cardiovascular, hepatic, or renal insufficiency; (6) patients with psychiatric disorders.

### Grouping of the patients

To ensure unbiased group allocation, patients were randomly assigned to either the esketamine or control group using a computer-generated random number sequence. Allocation concealment was maintained by using sealed, opaque envelopes prepared by an independent researcher not involved in patient recruitment or data analysis. This approach minimized selection bias and ensured that group assignments remained blinded until the intervention was assigned. The 150 patients meeting the inclusion criteria were randomly assigned into two groups using a random number table: the esketamine group (75 patients) and the control group (75 patients). The esketamine group consisted of 47 male and 28 female patients, with an age range of 45-71 years (mean age 54.76±8.94 years). In this group, 26 patients had stage I colon cancer, and 49 had stage II colon cancer. The control group consisted of 45 male and 50 female patients, aged 42-70 years (mean age 55.85±7.89 years), with 27 patients having stage I and 48 patients having stage II colon cancer. The baseline characteristics and perioperative data for all the enrolled patients was shown in Table 1.

**Table 1:** Baseline characteristic of first-trimester pregnant women. ASA: American Society of Anesthesiologists; MMSE: Mini-Mental State Examination

Item	Esketamine group (75)	Control group (75)	p-Value
Age (years)	54.76±8.94	53.83±7.89	0.867
Sex (male)	47 (62.7%)	45 (60.0%)	0.737
BMI (kg/m^2^)	22.87±2.05	23.04±2.15	0.784
ASA grading	60 (80.0%)	58 (77.3%)	0.972
ASA grading	15 (20.0%)	17 (22.7%)	
MMSE score	26 (25, 27)	26 (25, 27)	0.827
Operation duration (min)	175.8 (147.5, 206.8)	180.3 (152.3, 210.8)	0.302
Extubation time (min)	32.67±17.72	31.96±16.83	0.727
PACU stay time (min)	65.22±21.43	61.59±20.83	0.794

### Anesthesia protocol

All patients underwent general anesthesia. Induction was initiated with intravenous administration of 0.2-0.5 mg/kg etomidate, 0.5-1.0 μg/kg sufentanil, and 0.15-0.5 mg/kg cisatracurium. After tracheal intubation, anesthesia was maintained with continuous infusion of propofol at a rate of 4-12 mg/kg/h and remifentanil at 0.05-2 μg/kg/min.

For the esketamine group, following the induction of anesthesia, a bolus dose of 0.25 mg/kg esketamine was administered intravenously, followed by a continuous infusion at 0.12 mg/kg/h until the surgical incisions were closed. In contrast, the control group received an equivalent volume of normal saline intravenously as a substitute for esketamine.

### Biochemical and immunological analysis of serum and peripheral blood

Venous blood (10 mL) was collected pre- and postoperatively using sterile vacutainer tubes. For oxidative stress and inflammatory marker analysis, 5 mL of blood was centrifuged at 5,000 × g for 10 min at 4°C to isolate serum, which was aliquoted and stored at -80°C until assayed. The remaining 5 mL was anticoagulated with EDTA for immediate flow cytometric immune profiling.

Serum concentrations of glutathione (GSH; Cat# ab158881, Abeam), catalase (CAT; Cat# ab85464), malondialdehyde (MDA; Cat# ab118970), and superoxide dismutase (SOD; Cat# ab65554) were quantified using commercially available ELISA kits, following manufacturer protocols. Absorbance was measured at 450 nm using a Multiskan SkyHigh microplate reader (Thermo Fisher Scientific). Inflammatory cytokines—TNF-a (Human TNF-alpha Quantikine ELISA Kit, R&D Systems, Cat# DTA00C), CRP (CRP Human ELISA Kit, Invitrogen, Cat# BMS288INST), and IL-6 (Human IL-6 ELISA Kit, Abeam, Cat# ab46027)—were analyzed in duplicate, with intra- and inter-assay coefficients of variation <8%.

For immune profiling, EDTA-anticoagulated blood was stained with fluorochrome-conjugated monoclonal antibodies against CD5-FITC (Clone UCHT1), CD4-PE (Clone RPA-T4), and CD8-APC (Clone SK1) (BD Biosciences). Samples were incubated for 20 min in the dark, lysed using BD FACS Lysing Solution, and analyzed on a Beckman Coulter CytoFLEX flow cytometer. A minimum of 10,000 events per sample were acquired, and lymphocyte subsets were gated using CytExpert 2.4 software. The CD4+/CD8+ ratio was calculated to assess immune balance.

### Statistical analysis

All data were processed using SPSS 19.0 software (IBM, Armonk, NX USA). Continuous variables were expressed as mean±standard deviation (x̄±s), and comparisons between groups were made using the t-test. Categorical data were expressed as percentages and analyzed using the chi-square (χ^2^) test. A P value of less than 0.05 was considered statistically significant.

## Results

### Comparison of serum GSH and CAT levels before and after surgery

Prior to surgery, there were no significant differences in serum levels of glutathione (GSH) and catalase (CAT) between the esketamine and control groups (P > 0.05). However, after surgery, both groups showed a significant decrease in serum GSH and CAT levels compared to preoperative levels. Notably, the esketamine group demonstrated a more favorable change in serum GSH and CAT levels postoperatively, with significantly higher values compared to the control group (P < 0.05) (Table 2).

**Table 2:** Serum GSH and CAT levels before and after surgery. ^a^indicates P < 0.05, compared with 'before surgery'; ^b^indicates P < 0.05, compared with 'control group'.

Group	Time-point	GSH (mg/mL)	CAT (U/mL)
Control	Before Surgery	86.57 ±7.89	107.62±8.62
	After Surgery	60.16±5.57^a^	82.81 ±7.75^a^
Esketamine	Before Surgery	85.87±8.17	106.26±9.12
	After Surgery	72.45±6.65^a,b^	92.56±8.51^a,b^

### Comparison of serum MDA and SOD levels before and after surgery

Before surgery, there were no significant differences between the groups in terms of serum malon- dialdehyde (MDA) and superoxide dismutase (SOD) levels (P > 0.05). However, following surgery, both groups exhibited increased serum MDA levels and decreased SOD levels compared to preoperative values, indicative of enhanced oxidative stress. The esketamine group showed a more favorable postoperative change, with significantly lower MDA levels and significantly higher SOD levels compared to the control group (P < 0.05) (Table 3).

**Table 3:** Serum MDA and SOD levels before and after surgery. ^a^indicates P < 0.05, compared with 'before surgery'; ^b^indicates P < 0.05, compared with 'control group'.

Group	Time-point	MDA (mmol/L)	SOD (nU/mL)
Control	Before Surgery	5.25±0.51	99.49±10.05
	After Surgery	9.52±0.65^a^	69.95±7.05^a^
Esketamine	Before Surgery	5.59±0.46	98.44±9.72
	After Surgery	6.41 ±0.52^a,b^	84.55±8.02^a,b^

### Comparison of serum TNF-α, CRP, and IL-6 levels before and after surgery

Before surgery, there were no significant differences in the serum levels of tumor necrosis factor-α (TNF-α), C-reactive protein (CRP), and interleukin-6 (IL-6) between the two groups (P > 0.05). However, after surgery, both groups showed an increase in serum TNF-α, CRF) and IL-6 levels compared to preoperative values, indicating systemic inflammation and immune activation. The esketamine group had significantly lower postoperative levels of TNF-α, CRF) and IL-6 compared to the control group, demonstrating a more favorable anti-inflammatory response (P < 0.05) (Table 4).

**Table 4:** Serum TNF-α, CRP, and IL-6 levels before and after surgery. ^a^indicates P < 0.05, compared with 'before surgery'; ^b^indicates P < 0.05, compared with 'control group'.

Group	Time-point	TNF-α (pg/mL)	CRP (mg/L)	IL-6 (pg/mL)
Control	Before Surgery	25.98±3.05	5.56±1.02	17.23±2.39
	After Surgery	54.37±5.80^a^	18.76±2.06^a^	82.38±8.46^a^
Esketamine	Before Surgery	27.03±3.23	5.61 ±0.88	18.07±3.02
	After Surgery	40.32±4.84^a,b^	12.29±1.79^a,b^	50.83±5.05^a,b^

### Comparison of peripheral blood T lymphocyte subsets before and after surgery

There were no significant differences in the levels of peripheral blood CD3^+^, CD4^+^, and CD8^+^ T lymphocytes or the CD4^+^/CD8^+^ ratio between the two groups before surgery (P > 0.05). However, postoperatively, the levels of CD3^+^ and CD4^+^ T lymphocytes, as well as the CD4^+^/CD8^+^ ratio, decreased, while the CD8^+^ T lymphocyte levels increased in both groups. Notably, the esketamine group showed a more favorable postoperative immune response, with significantly higher levels of CD3^+^ and CD4^+^ cells and a more balanced CD4^+^/CD8^+^ ratio compared to the control group (P < 0.05) (Table 5).

**Table 5:** Peripheral Blood T Lymphocyte Subsets Before and After Surgery. ^a^indicates P < 0.05, compared with 'before surgery'; ^b^indicates P < 0.05, compared with 'control group'.

Group	Time-point	CD3^+^ (%)	CD4^+^(%)	CD8^+^(%)	CD4^+^/CD8^+^
Control	Before Surgery	55.72±6.34	35.34±3.45	22.75±2.04	1.57±0.15
	After Surgery	37.05±4.92^a^	19.78±2.09^a^	27.68±2.67^a^	0.72±0.09^a^
Esketamine	Before Surgery	55.49±5.87	34.98±3.29	22.53±2.13	1.56±0.18
	After Surgery	45.18±5.01^a,b^	26.51 ±2.76^a,b^	23.55±2.22^a,b^	1.12 ±0.12^a,b^

## Discussion

The pathogenesis of colorectal cancer (CRC) remains incompletely understood, although several factors such as genetic predisposition, high-fat diets, and inadequate fiber intake have been implicated in its development [18] [19]. Laparoscopic surgery, known for its minimally invasive nature, has become the preferred surgical approach for colorectal cancer due to its advantages in reducing trauma and promoting faster recovery. However, it still presents perioperative stress injuries, which can complicate the postoperative period. Perioperative stress responses can disrupt immune function, lead to abnormal release of cytokines and cortisol, and trigger a cascade of inflammatory responses, all of which may negatively affect postoperative recovery and clinical outcomes [20]. Clinically, remifentanil-based anesthesia is commonly used to mitigate stress responses during surgery, though it is associated with risks such as postoperative agitation and pain hypersensitivity [21].

Esketamine, a more potent enantiomer of ketamine, has recently gained attention for its anesthetic and analgesic properties [22]. It exerts its effects through NMDA receptor antagonism, which not only induces anesthesia but also possesses antidepressant and anti-inflammatory effects [23]. Unlike ketamine, esketamine offers advantages in terms of faster recovery, lower incidence of psychotropic side effects, and enhanced tolerance.

This study investigated the effects of esketa- mine-based anesthesia on serum levels of oxidative stress markers (GSH, CAT, MDA, SOD), inflammatory cytokines (TNF-α, CRF) IL-6), and T lymphocyte subsets in patients undergoing laparoscopic colorectal cancer surgery, with the aim of reducing postoperative stress-related damage. Our findings demonstrate that, compared to preoperative values, both groups showed significant reductions in GSH, CAT, and SOD levels, alongside increased MDA levels after surgery. However, the esketamine group exhibited significantly better postoperative preservation of GSH, CAT, MDA, and SOD levels, suggesting a better antioxidant response (P < 0.05).

Laparoscopic surgery requires the establishment of pneumoperitoneum, which can cause ischemia in abdominal organs during the procedure. The subsequent reperfusion after surgery may lead to oxidative stress and damage, characterized by a decrease in antioxidant activity (GSH, CAT, SOD) and an increase in oxidative products such as MDA. Esketamine has been shown to enhance the body's antioxidant capacity, helping to alleviate oxidative stress-induced damage. In this study, esketamine-based anesthesia was associated with a significantly more favorable postoperative oxidative stress profile, supporting its potential role in mitigating oxidative stress during laparoscopic colorectal cancer surgery.

Additionally, serum levels of pro-inflammatory cytokines (TNF-α, CRF) IL-6) were elevated in both groups after surgery, reflecting the inflammatory response triggered by surgical trauma. The esketamine group demonstrated significantly lower levels of these cytokines compared to the control group, indicating that esketamine effectively attenuates the inflammatory response (P < 0.05). Inflammation, a key component of the stress response, is known to exacerbate tissue damage following surgery. Previous studies have suggested that esketamine can suppress the overproduction of inflammatory mediators, thereby reducing tissue injury and improving recovery.

Moreover, the study also assessed the impact of esketamine on cellular immunity by analyzing peripheral blood T lymphocyte subsets. While both groups showed a decrease in CD3^+^ and CD4^+^ T cell levels and an increase in CD8^+^ T cells postoperatively, the esketamine group had more favorable changes in these immune parameters, with significantly higher CD3^+^ and CD4^+^ T cell counts and a more balanced CD4^+^/CD8^+^ ratio compared to the control group (P < 0.05). These findings suggest that esketamine-based anesthesia may help preserve immune function during the perioperative period, which is critical for enhancing recovery and preventing infections postsurgery.

### Novelty and clinical implications

The novelty of this study lies in its exploration of esketamine-based anesthesia specifically in the context of laparoscopic colorectal cancer surgery. While the effects of esketamine on oxidative stress and inflammation have been investigated in other surgical settings, our study is one of the first to examine these effects in the context of laparoscopic colorectal cancer resection. The results suggest that esketamine- based anesthesia offers a promising strategy to mitigate the oxidative and inflammatory responses that typically accompany major surgeries, thereby improving postoperative recovery.

Clinically, the findings support the potential of esketamine as a valuable adjunct to anesthesia protocols for laparoscopic colorectal cancer surgery. Its ability to reduce oxidative stress and inflammation, while preserving immune function, may translate into improved outcomes, such as reduced postoperative complications and faster recovery times. Given these advantages, esketamine may be considered a beneficial anesthetic option for patients undergoing minimally invasive colorectal cancer surgery.

### Limitations of the study

While our study provided valuable insights into the effects of esketamine-based anesthesia on oxidative stress, inflammation, and immune function, several limitations should be acknowledged. First, the sample size, though adequate for statistical significance, may have limited the generalizability of our findings. A larger, multi-center trial would have been necessary to confirm these results across diverse patient populations. Second, inter-patient variability in immune response may have influenced our findings. Factors such as preoperative nutritional status, genetic predisposition, and comorbid conditions could have impacted oxidative stress levels and immune modulation. Future studies should have incorporated stratified analyses or subgroup assessments to account for these variables and better understand patient-specific responses to esketamine-based anesthesia. Finally, our study primarily focused on short-term postoperative outcomes. Long-term follow-up would have been needed to determine whether esketamine's immunomodulatory effects translated into improved clinical outcomes, including reduced postoperative complications and enhanced recovery in colorectal cancer patients.

Esketamine's ability to reduce oxidative stress, suppress inflammation, and preserve immune function makes it a promising addition to perioperative care in colorectal cancer surgery. Its NMDA receptor antagonism and analgesic effects may also reduce opioid consumption, potentially minimizing immunosuppression and aiding recovery. Compared to conventional anesthetics like propofol and remifentanil, esketamine uniquely modulates immune function and mitigates perioperative stress. It has been shown to decrease oxidative stress markers and inflammatory cytokines such as TNF-α, CRF) and IL-6, promoting better postoperative recovery in laparoscopic colorectal cancer resection. Additionally, esketamine's role in maintaining T lymphocyte subsets may lower the risk of postoperative infections, particularly in elderly or immunocompromised patients. Given these benefits, integrating esketamine into routine perioperative protocols could enhance surgical outcomes. Future guidelines should consider its inclusion in enhanced recovery after surgery (ERAS) strategies to optimize patient care.

## Conclusion

In conclusion, esketamine-based anesthesia appears to significantly reduce postoperative oxidative stress and inflammatory responses in patients undergoing laparoscopic colorectal cancer surgery. It also seems to preserve cellular immune function, making it a promising anesthetic option for this patient population. Given its beneficial effects on perioperative outcomes, esketamine may be a valuable tool in improving recovery and reducing complications in colorectal cancer surgeries. Further research with larger sample sizes and long-term follow-up is needed to confirm the clinical benefits of esketamine in this setting.

## Dodatak

### Acknowledgements

This work was supported by the Research Project of Jiangsu Provincial Health Commission (NO.M2021058).

### Conflict of interest statement

All the authors declare that they have no conflict of interest in this work.
